# Multi-susceptibility genes associated with the risk of the development stages of esophageal squamous cell cancer in Feicheng County

**DOI:** 10.1186/1471-230X-11-74

**Published:** 2011-06-14

**Authors:** Qing Da Li, Hao Li, Mei Shu Wang, Tao Yu Diao, Zhi Ying Zhou, Qiang Xue Fang, Fang Yan Yang, Qing Hui Li

**Affiliations:** 1 The Key Laboratory of Cardiovascular Remodeling and Function Research, Chinese Ministry of Education and Chinese Ministry of Health, Qilu Hospital of Shandong University, 107# Wenhuaxi Road, Jinan, 250012, China; 2 Department of Hematology of Tumor Center, Qilu Hospital of Shandong University, 107# Wenhuaxi Road, Jinan, 250012, China; 3 Department of Epidemiology, College of Public Health of Shandong University, 104# Wenhuaxi Road, Jinan, 250012, China; 4 Department of Epidemiology, Institute of Basic Medicine of Shandong Academy of Medical Sciences, 18877# Jingshi Road, Jinan, 250062,China; 5 Department of Statistics, School of Economics, Shandong University, 27# Shada Nanlu, Jinan, 250100,China; 6 Department of Infectious Disease of Shandong Center for Disease Control and Prevention, 72# Jingshi Road, Jinan, 250014, China; 7 Department of Epidemiology, College of Public Health of Southwestern University, 16# Renminnan Road, Chengdou, 610041, China

## Abstract

**Background:**

The purpose of this study was to evaluate the association of multi-genotype polymorphisms with the stepwise progression of esophageal squamous cell cancer (ESCC) and the possibility of predicting those at higher risk.

**Methods:**

A total of 1,004 subjects were recruited from Feicheng County, China, between Jan. 2004 and Dec. 2007 and examined by endoscopy for esophageal lesions. These subjects included 270 patients with basal cell hyperplasia (BCH), 262 patients with esophageal squamous cell dysplasia (ESCD), 226 patients with ESCC, and 246 controls with Lugol-voiding area but diagnosed as having normal esophageal squamous epithelial cells by histopathology. The genotypes for *CYP2E1 *G1259C, *hOGG1 *C326G, *MTHFR *C677T, *MPO *G463A, and *ALDH2 *allele genes were identified in blood samples collected from all participants.

**Results:**

The alleles *ALDH2 *and *MTHFR *C677T were critical for determining individual susceptibility to esophageal cancer. Compared to the *ALDH *1*1 genotype, the *ALDH *2*2 genotype was significantly associated with increased risks of BCH, ESCD, and ESCC. However, the TT genotype of *MTHFR *C677T only increased the risk of ESCC. Further analysis revealed that the combination of the high-risk genotypes 2*2/1*2 of *ALDH *2 and TT/TC of *MTHFR *C677T increased the risk of BCH by 4.0 fold, of ESCD by 3.7 fold, and ESSC by 8.72 fold. The generalized odds ratio (OR_G_) of the two combined genotypes was 1.83 (95%CI: 1.55-2.16), indicating a strong genetic association with the risk of carcinogenic progression in the esophagus.

**Conclusions:**

The study demonstrated that the genotypes ALDH2*2 and *MTHFR *677TT conferred elevated risk for developing esophageal carcinoma and that the two susceptibility genotypes combined to synergistically increase the risk.

## Background

Esophageal cancer is the fourth most common cause of cancer-related death in China. Esophageal cell carcinoma (ESCC) is by far the most common subtype of esophageal cancer, followed distantly by adenocarcinoma, which accounts for less then 3% of all esophageal cancers in high incidence areas of China [1].

The pathogenesis of ESCC is thought to include a stepwise progression from basal cell hyperplasia (BCH) to low-grade dysplasia (LGD), high-grade dysplasia (HGD), carcinoma *in situ*, and finally invasive carcinoma. Japanese and Chinese pathologists prefer to classify LGD as mild-dysplasia or moderate-dysplasia, and HGD as severe-dysplasia. They defined esophageal dysplasia as a precancerous lesion of ESCC [2-5]. Epidemiological studies indicated that esophageal dysplasia was associated with a significantly increased risk of developing invasive ESCC [6-10].

Endoscopic screening with the Lugol dye method combined with pathologic evaluation has proven useful in screening for early esophageal cancer and in ascertaining the different stages of esophageal carcinogenesis in high-incidence areas [11]. This test is relatively expensive, however, and patients may experience pain and discomfort, so only a small percentage of the population may be willing to participate in this testing program. With the development of molecular techniques, however, it is now possible to employ useful biomarkers to identify subjects at high-risk who should receive this pathological screening test.

Recent studies have suggested that a variety of genes may be associated with susceptibility to ESCC. These include the aldehyde dehydrogenase-2 gene (*ALDH2*) [12-14], the methylene tetrahydrofolate reductase gene (*MTFHR*) [15-17], cytochrome P450 2E1 (CYP2E1) [18,19], myeloperoxidase gene (*MPO*) [20,21], and the human 8-oxoguanine glycosylase 1 gene (*hOGG1*) [22]. Indeed, there was a weak association between each of these five susceptibility genes alone and esophageal cancer. It is unclear, however, whether a combination of these susceptibility genes could be employed as useful biomarkers to predict significantly elevated risk for ESCC.

In the present study, we developed a program for evaluation of esophageal lesions using endoscopic screening with the Lugol dye method. The screening ran from Jan. 2005 to Dec. 2007 in Feicheng County, China. In addition to endoscopic evaluation for various types of esophageal lesions, the multi-genotype polymorphisms of *CYP2E1 *G1259C, *hOGG1 *C326G, *MTHFR *C677T, *MPO *G463A, and *ALDH2 *genes were identified for each individual subject. Therefore, this program provided a valuable opportunity to first examine the potential association between a specific combination of genotypes and the carcinogenic progression during ESCC, and then to evaluate the possibility of using the genotype combination as a biomarker to predict ESCC risk.

## Methods

### Study subjects

The human subject protocol for this study was approved by the Ethics Committee of the Medical Faculty of the Shandong Academy of Medical Sciences. Written informed consent was obtained from all participants.

For subject recruitment, a questionnaire interview was first conducted to collect information, such as sociodemographic characteristics, alcohol intake, tobacco use, and family history of esophageal carcinoma. Then, a routine physical examination, electrocardiogram, and abdomen ultrasound were provided to all candidates. Those who had liver diseases, cardiovascular diseases, lung diseases, or head-and-neck diseases were excluded for further consideration. All candidate subjects were given an endoscopic staining examination with 1.2% iodine solution for evaluation of esophageal lesions. Furthermore, for persons with a non-staining area of the mucosa, random 4-quadrant biopsy specimens were obtained at 2-cm intervals. Specimens were processed by the standard procedure [5] and histopathological diagnoses were made by two independent pathologists.

A total of 10125 persons took part in the endoscopic staining examination. Of these patients, 1364 had a non-staining area of the mucosa and were diagnosed by histopathologic analysis of biopsy tissue. These patients included 280 with basal cell hyperplasia (BCH), 577 with esophageal squamous cell dysplasia (ESCD), 94 with esophageal squamous cell cancer (ESCC) at early stage, and 267 controls with normal esophageal squamous epithelial cells. We randomly selected 50% of the ESCD for further study using the program SPSS. Another 146 patients with ESCC were referred from the Hospital of Feicheng County. In total, 1004 subjects with pathological data were available for the analysis, including 270 patients with BCH, 262 patients with ESCD, 226 patients with ESCC, and 246 controls with Lugol-voiding area but diagnosed as normal by histopathology of esophageal squamous epithelial cells. For genotype assays, 5-8 ml of blood were collected from each participant in a sterile cryogenic vial and stored at -70 ºC until processed.

The sample size was calculated by the Power 3.0 software (http://dceg.cancer.gov/bb/tools/power). We recalculated the sample size based on the following parameters: design: case-control study; case: control = 1:1; probability of esophageal cancer = 0.001 for the aged 40-69; observed minor allele frequency 0.1-0.25; a moderate genetic risk effect (OR = 1.2-1.3); P-value = 0.05; 1-β = 0.8; additive effect model OR = 3.0 of two genes exposure, the sample size = 265. According to the actual sample sizes in the study, the posterior powers (1-β), were 0.735, 0.796, 0.808 and 0.769 for the above four groups of ESCC, ESLD, BCH and controls, respectively.

### PCR-RFLP Polymorphism for *MTHFR *C677T, *MPO *G463A and *CYP2E1 *G1259C

The PCR reaction was carried out in gradient PCR instrument (Eppendorf, Germany). The reaction mixture contained 25 ng DNA, 10 mM dNTP 0.5 μl, 10 × PCR Buffer 2.5 μl, 10uM of each primer (Table 1) and 0.5 units of Taq DNA polymerase with the buffer (20 mmol/L Tris-HCl, pH 8.4; 50 mmol/L KCl) in a volume of 25 μl.

**Table 1:** PCR conditions for genotypes of test DNA genes in the study

Gene	Primer sequence	Annealing	Restriction enzyme
*MTHFR* C677T	F: 5'-TGA AGG AGA AGG TGT CTG CGG GA-3' R: 5'-AGG ACG GTG CGG TGA GAG TG-3'	62°C	*Hin*f I
*CYP2E1* G1259C	F: 5'-CCA GTC GAG TCT ACA TTG TCA-3' R: 5'-TTC ATT CTG TCT TCT AAC TGG-3'	55°C	*PstI*
*hOGG1* C326G	F: 5'-GGA AGG TGC TTG GGG AAT-3' R: 5'-ACT GTC ACT AGT CTC ACC AG-3'	58°C	*Fnu4H*I
*MPO* G463A	F: 5'-CGG TAT AGG CAC ACA ATG GTG AG-3' R: 5'-GCA ATG GTT CAA GCG ATT C-3'	62°C	*AciI*
*ALDH2*	F1: 5'-TCA TGC CAT GGC AAC TCC AGC-3' R1: 5'-CCC ACA CTC ACA GTT TTC TCT TC-3' F2: 5'-TAC GGG CTG CAG GCA TAC ACT A-3 R2: 5'-TGA TCC CCA GCA GGT CCT GAA-3'	60°C	

PCR conditions were 95°C for minutes, followed by 35 cycles of 95°C for 60 seconds optimal annealing temperature × °C (Table 1) for 60 seconds and 72°C for 60 seconds followed by a final extension step of 72°C for 7 minutes.

The 5 μl PCR product of *MTHFR *C677T gene was digested by a *Hin*fI restriction enzyme. Digestion products were visualized after electrophoresis on a 3% agarose gel with ethidium bromide. Wild types (677CC) produced a single band at 198bp. Heterozygotes (677CT) produced 198, 175, and 23bp fragments. Homozygous mutants (677TT) produced 175 and 23bp fragments [16].

The PCR product of the *MPO *G463A gene digested by an *AciI *restriction enzyme yielding three possible genotypes which were defined by three distinct banding patterns: A/A 289 and 61bp fragments, A/G 289, 169, 120, and 6lbp fragments, and G/G 169, 120, and 6lbp fragments [20].

The PCR product of *CYP2E1 *G1259C gene was digested with *Pst*I restriction endonuclease for 8 hours at 37°C. Three possible genotypes were defined by three distinct banding patterns: C1/C1 410bp fragment, C1/C2 410, 290 and 120 bp fragments, and C2/C2 290- and 120-bp fragments [18].

### PCR for *ALDH2*

The two pairs of primers shown in Table 1 were used in the PRC assay. F1 and R1 were used to amplify the ALDH2*1 allele (296bp), and F2 and R2 to amplify the ALDH2*2 allele (203bp). Two 25 μL reaction tubes were needed for each specimen to amplify ALDH2*1 (G) and ALDH2*2 (A) respectively, each containing 100 ng DNA, 0.12 mmol/L dNTPs, 12.5 pmol F1 (or R1) primer, 12.5 pmol F2 (or R2) primer, 0.5 U *Taq *polymerase, and 2.5 μL 10 × PCR buffer (containing 15 mmol/L MgCl2). The reaction tubes were heated to 95°C for 5 min followed by 30 cycles of 95°C for 60 s, 60°C for 60 s, 72°C for 60 s, and 72°C for 45 s, and then followed by a final extension of 5 min at 72°C. 10 μL PCR products were used in agarose gel electrophoresis and the electrophoresis result was photographed.

Two lanes were used for each specimen. If one showed 296 bp band and the other showed no band, the corresponding genotype was ALDH2*1/2*1 (G/G); if one showed 296 bp band and the other showed 203 bp band, the corresponding genotype was ALDH2*1/2*2 (G/A); and if one showed 203 bp band and the other showed no band, the corresponding genotype was ALDH2*2/2*2 (A/A) [23].

### PCR-SSCP analysis of *hOGG1 *C326G

The PCR product was denatured with formamide at 95°C for 15 min, quenched on ice, and loaded to polyacrylamide gels under several conditions. Visualization was performed with a silver stain kit (Wako, Osaka, Japan) as described [22]. The PCR product of *hOGG1 *C326G gene is digested by *Fnu4H*I on the polyacrylamide gels showed C/C genotype is a band at 200bp, G/G genotype is a band at 100bp, and C/G genotype is two bands at 200bp and 100bp.

### Quality Control

The genotypes of the DNA samples were identified without knowledge of the case or control status; 5% were randomly selected as a sample set of cases and controls that were genotyped by different investigators, and the reproducibility was 100%. Each PCR procedure was performed with a blank control (without DNA template) and positive and negative controls. Cycle sequencing PCR product was performed to confirm the accuracy of this method of single-nucleotide polymorphisms (SNP) identification. When any of these controls failed, the PCR was repeated for the batch of samples.

### Statistical Analysis

Pearson's Chi-Square and Kruskal-Wallis H tests were used to examine differences in sociodemographic characteristics, alcohol intake, tobacco use, and family history of esophageal cancer among the four diagnostic groups (Control, BCH, ESCD, and ESCC). Smoking index represents the number of cigarettes per day multiplied by the years of smoking. Alcohol drinking index equals the amount of alcohol consumed per month multiplied by drinking years. Allele frequencies were calculated using allele counting tests for Hardy-Weinberg equilibrium and were analyzed by the Chi-square test. Odds ratios (ORs) and 95% confidence interval (95%CI) were calculated in the multinomial Logistic model after adjusting for the variables of age, smoking index, and drinking index.

An additional analysis based on the generalized odds ratio (OR_G_) was also performed. The OR_G _utilizes the complete genotype distribution and provides an estimate of the magnitude of the association between disease status and genotype [24]. The OR_G _and 95%CI were calculated using the software "ORGGASMA" (downloaded from http://biomath.med.uth.gr). All other statistical analysis were performed using SPSS (version 15.0), and *P <*0.05 (two-sided) was accepted as statistically significant.

## Results

### Characteristics of demographic and selected variables

Demographic characteristics and selected variables are shown in Table 2. All seven variables, including gender, age, school years, income per year, smoking index, alcohol drinking status, and family history of esophageal cancer were significantly different among the four groups. Each variable also has a significant linear by linear association with the carcinogenic stages in transition from normal esophageal mucosa to carcinoma.

**Table 2:** Distribution of selected variables in the BCH, ESCD, ESCC and controls

Feature	BCH (n = 270)	ESCD (n = 262)	ESCC (n = 226)	Controls (n = 246)	*x^2^(P^c^)*
Gender (n,%)					
Male	185(68.5)	151 (57.7)	162 (71.7)	125(50.8)	29.062
Female	85(31.5)	111 (42.3)	64 (28.3)	121(49.2)	(<0.001)
Age(yr) (n,%)					
<50	70(26.0)	40(15.3)	38(16.8)	142(57.7)	150.471
50-	138(51.1)	138(52.7)	102(45.1)	66(26.8)	(<0.001)
≥60	62(23.0)	84(32.1)	86(38.1)	38(15.4)	
School (yr) (n,%)					
≤6	110 (40.9)	148 (56.5)	144 (63.7)	102(41.5)	39.792
9	129(47.7)	92 (35.1)	72(31.9)	120(48.8)	(<0.001)
≥10	31(11.5)	22 (8.4)	10(4.4)	24(9.8)	
Income per year ($) (n,%)					
<150	129(47.7)	134 (51.1)	98 (43.4)	164(66.7)	36.494
150-	117(43.4)	92(35.1)	94(41.6)	66(26.8)	(<0.001)
≥350	24(8.9)	36(13.7)	34(15.0)	16(6.5)	
Smoking index^a^(n,%)					
None	109 (40.4)	148 (56.9)	80 (35.4)	148(60.2)	60.087
<450	63 (23.4)	38(14.6)	36 (15.9)	48(19.5)	(<0.001)
≥450	98 (36.2)	74(28.5)	110 (48.7)	50(20.3)	
Alcohol drinking Status^b^(n,%)					
None	114 (42.4)	136 (53.1)	76 (33.6)	138(56.1)	68.712
<100	43(16.0)	44(17.2)	32 (14.2)	62(25.2)	(<0.001)
≥100	112(41.6)	76(29.7)	118 (52.2)	46(18.7)	
Family history of esophagus cancer (n,%)					
None	231 (85.5)	2021(77.1)	190(84.1)	222(90.2)	17.027
yes	39 (14.5)	60(22.9)	36(15.9)	24(9.8)	(<0.001)

^a ^smoking index = cigarette/day × number of smoking years.^b ^alcohol drinking ≥100g/day means heavy drinker. ^c ^*P *: Chi-square test was for proportions among the four groups.

*Abbreviations: *BCH, Basal cell hyperplasia; ESCD, esophageal squamous cell dysplasia; ESCC, Esophageal Squamous cell cancer.

### Associations of *CYP2E1 *G1259C, *MPO *G463A, *MTHFR *C677T, *hOGG1 *C326G, and *ALDH2 *genotypes with BCH, ESCD, and ESCC

The frequency distribution of *CYP2E1 *G1259C, *MPO *G463A, *MTHFR *C677T, *hOGG1 *C326G, and *ALDH2 *genotypes are shown in Table 3. The Hardy-Weinberg test for the control group showed that the distributions of the five genotypes were in equilibrium.

**Table 3:** Distribution of CYP2E1, MPO, MTHFR and ALDH2 genotypes in the BCH, ESCD, ESCC and controls, n(%)

Feature	BCH	ESCD	ESCC	Controls	*x^2^&P^a^*
*CYP2E1 *G1259C					
C2/C2	4(1.5)	0	0	11(4.5)	1.519
C1/C2	83(30.8)	73(27.9)	67(29.6)	62(25.0)	0.678b
C1/C1	183(67.7)	189(72.1)	159(70.4)	173(70.5)	
*MPO *G463A					
G/G	221(81.8)	234(89.2)	175(77.4)	191(77.8)	15.942
A/G	49(18.2)	28(10.8)	44(19.4)	52(21.2)	0.001 ^b^
A/A	0	0	7(3.2)	2(1.0)	
*hOGG1 *C326G					
G/G	43(15.9)	26(10.1)	13(5.9)	26(10.4)	24.003
C/G	123(45.7)	160(60.9)	126(55.9)	123(50.0)	<0.001
C/C	104(38.4)	76(29)	86(38.2)	97(39.6)	
*MTHFR *C677T					
T/T	45(16.7)	85(32.4)	64(28.4)	58 (23.6)	20.705
C/T	113(41.9)	82(31.3)	85(37.5)	97 (39.4)	0.002
C/C	112(41.4)	95(36.3)	77(34.1)	91 (37.0)	
*ALDH2*					
G/G(2*2)	106(39.4)	145(55.3)	76(33.6)	41(16.7)	80.423
A/G(1*2)	134(49.5)	85(32.5)	129(57.1)	164(66.6)	<0.001
A/A(1*1)	30(11.1)	32(12.2)	21(9.3)	41(16.7)	

^a^*P *: Chi-square test was for proportions among the four groups. ^b ^: Kruskal-Wallis H test. *Abbreviations: *BCH, Basal cell hyperplasia; ESCD, esophageal squamous cell dysplasia; ESCC, Esophageal Squamous cell cancer.

As shown in Table 4 after adjusting for the potential confounders gender, age, school years, income per year, smoking index, alcohol drinking status, and family history of esophageal cancer, we found that polymorphism of the *ALDH2 *genotype was associated with BCH, ESCD, and ESCC. Compared to the *ALDH *1*1 genotype, the *ALHD *2*2 genotype was associated with significantly increased risks of BCH, ESCD, and ESCC (with the adjusted OR = 4.15, 95% CI, 2.33-7.40 for BCH, OR = 4.54, 95% CI 2.32-8.89 for ESCD, and OR = 3.38, 95% CI 1.64-6.95 for ESCC). Furthermore, the TT genotype of *MTHFR *C677T increased the relative risk in the ESCC group, while the GG genotype of *hOGG1 *C326G increased the risk in the ESCD group.

**Table 4:** Risk genotypes related to BCH, ESCD and ESCC in the multinomial logistic regression models ^a^

Factors	BCH OR(95%CI)	ESCD OR(95%CI)	ESCC OR(95%CI)	OR_G_(95%CI) #
*MTHFR *C677T				
TT	0.91(0.59-1.40)	0.78(0.48-1.28)	1.85(1.02-3.34)	1.16(1.00-1.35)
CT	0.89(0.58-1.37)	0.56(0.34-1.03)	1.72(0.95-3.10)	
TT&TC	0.90(0.62-1.32)	0.66(0.43-1.12)	1.71(1.01-2.91)	
CC	1.00	1.00	1.00	
*MPO *G463A				
GG	1.09(0.64-1.86)	2.13(0.90-5.08)	0.96(0.52-1.78)	0.93(0.72-1.19)
GA&AA	1.00	1.00	1.00	
*CYP2E1 *G1259C				
C2/C2 & C1/C2	1.15(0.64-2.09)	0.79(0.38-1.62)	1,01(0.43-2.33)	0.94(0.77-1.14)
C1/C1	1.00	1.00	1.00	
*hOGG1 *C326G				
GG	1.56(0.71-3.43)	1.23(0.46-3.26)	0.50(0.14-1.81)	1.00(0.85-1.78)
GC	1.10(0.66-1.86)	2.33(1.24-4.36)	1.45(0.72-2.92)	
GG&GC	1.2990.73-1.97)	2.08(1.14-3.76)	1.23(0.63-2.40)	
CC	1.00	1.00	1.00	
*ALDH2*				
2*2	4.15(2.33-7.40)	4.54(2.32-8.89)	3.38(1.64-6.95)	1.52(1.30-1.77)
1*2	1.19(0.72-1.95)	0.65(0.34-1.22)	1.39(0.73-2.64)	
2*2&1*2	1.73(1.07-2.81)	1.43(0.79-2.58)	1.80(0.97-3.38)	
1*1	1.00	1.00	1.00	

^a^: Adjusted for age, sex, income, school year, smoking, drinking and family history of esophageal cancer. *Abbreviations: *BCH, Basal cell hyperplasia; ESCD, esophageal squamous cell dysplasia; ESCC, Esophageal Squamous cell cancer; OR_G_: generalized odds ratio.

#: OR_G_: calculated by the software "ORGGASMA" of the web site htpp://biomath.med.uth.gr.(note:: added 0.5 to zero frequency of cell).

Based on the values of OR_G_, only the *MTHFR *C677T genotype (OR_G _= 1.16; 95%CI: 1.00-1.35) and *ALDH *2 (OR_G _= 1.52; 95%CI: 1.30-1.77) genotype showed significant genetic association with the risk of carcinogenic progression of the esophagus.

### Combing two-susceptibility genotypes analysis

Subjects with either homozygous or heterozygous variant alleles (2*2 or 1*2) of *ALDH *2 had increased risk of developing BCH, ESCD, and ESCC compared to those who had wild type *ALDH *2 (Table 4). Furthermore, TT and CT genotypes of *MTHFR *C677T were found to enhanced susceptibility to ESCC compared to the CC genotype.

The frequencies of the various combinations of the susceptible genotypes of *ALDH *2 and *MTHFR *C677T genes were calculated and analyzed for their associated risks of diseases (Tables 5 and 6). The OR values for the associations of the combined susceptibility genotypes with esophageal lesions was significantly higher than for the individual genotypes (Table 6). For example, after adjusting for the aforementioned seven potential confounders, the combinations of *ALDH *2 2*2/1*2 and *MTHFR *TT/TC genotypes were associated with significantly increased risks for BCH, ESCD, and ESCC compared to patients with the 1*1 *ALDH *2 and CC *MTHFR *C677T genotype. The ORs (95%CI) were 4.03(2.14-7.57) for BCH, 3.70(1.74-7.87) for ESCD, and 8.72(3.24-23.48) for ESCC. Furthermore, the OR_G _of the two combined genotypes was 1.83(95%CI: 1.55-2.16), indicating a significant genetic association with the risk of carcinogenic progression in the esophagus.

**Table 5:** Distribution of subjects with the number of susceptibility of the combination of the *ALDH2 *and *MTHFR *genes in the four groups

ALDH2	MTHFR	ESCC n,%	ESCD n,%	BCH n,%	Normal n,%
2*2/1*2	TC/TT	163(72.1)	138(52.7)	150(55.5)	100(40.7)
2*2/1*2	CC	38(16.8)	84(32.1)	80(29.6)	54(22.0)
1*1	TC/TT	17(7.5)	26(9.9)	25(9.3)	52(21.1)
1*1	CC	7(3.1)	14(5.3)	15(5.6)	40(16.2)

Total		226 (100.0)	262(100.0)	270(100.0)	246(100.0)

**Table 6:** ORs (95%CI) of the susceptibility genotypes of the combination of the *ALDH2 *and *MTHFR *genes related to lesions of esophagus ^a^

		Crude OR(95%CI)			Adjusted OR(95%CI)		
		
ALDH2	MTHFR	BCH	ESCD	ESCC	BCH	ESCD	ESCC
2*2/1*2	TC/TT	4.00 (2.28-7.17)	3.92 (2.06-8.11)	9.31 (3.81-23.12)	4.03 (2.14-7.57)	3.70 (1.74-7.87)	8.72 (3.24-23.48)
2*2/1*2	CC	3.95 (2.16-7.42)	4.44 (2.25-9.60)	4.02 (1.58-10.94)	4.21 (2.14-8.29)	4.68 (2.11-10.38)	4.45 (1.54-12.87)
1*1	TC/TT	1.37 (0.65-2.50)	1.43 (0.67-3.29)	1.87 (0.68-5.45)	1.18 (0.56-2.49)	1.23 (0.51-2.95)	1.80 (0.57-5.68)
1*1	CC	1.00	1.00	1.00	1.00	1.00	1.00

^a^: Adjusted for age, sex, income, school year, smoking, drinking and family history of esophageal cancer. *Abbreviations: *BCH, basal cell hyperplasia; ESCD, esophageal squamous cell dysplasia; ESCC, Esophageal Squamous cell cancer.

## Discussion

Feicheng County has a high incidence of esophageal cancer compared to the rest of China. Worldwide mortality rates have decreased from 75.82 per 100,000 in 1970-1974 to 57.22 per 100,000 in 2000-2004 [25]. In the present study, we demonstrate that specific allelic combination significantly increased the risk for esophageal cancer (by as much as 8-fold). While reproducibility of studies linking genotype to disease risk is often problematic, there are several strengths of this study. First, the subjects in the study were diagnosed by biopsy, so misclassification bias was very low. Our DNA collection method avoided biases that may arise from single-center or multi-center collection. Furthermore, several steps were taken to ensure high quality and repeatability of results. These included initial DNA sequencing of SNP regions to prove the reliability, blinding of the operator to the case-control status of samples to reduce observer bias, and simultaneous analysis of case and control samples to avoid differential misclassification. Moreover, the allele frequencies reported among normal controls in this study were similar to those reported in previous studies of Chinese subjects. In sum, these controls indicated that our findings have high validity and reliability.

In the present study, we found that the *ALDH2 *genotype was associated with BCH, ESCD, and ESCC, the main stages of carcinogenic transition in the esophagus. Acetaldehyde is formed by the oxidation of ethanol by alcohol dehydrogenase (*ADH*), and is eliminated by aldehyde dehydrogenase (*ALDH*). The *ALDH2 *gene carries two alleles, ALDH2*1 and ALDH2*2, with different kinetic properties and distinct distributions among ethnicities [23,26]. The ALDH2*2 allele is found at a frequency of only 50% in Orientals, while the ALDH2*1 allele is more predominant in Caucasians [26]. The ALDH2*2 allele codes for an inactive *ALDH2*, and is closely associated with alcohol related cancers in the upper aerodigestive tract [27-29]. The accumulation of acetaldehyde plays a protective role against excessive alcohol consumption as it causes unpleasant reactions, including "Oriental facial flushing" and other symptoms due to alcohol sensitivity, such as headache, nausea, vomiting, tachycardia, hypotension, and sleepiness [30]. Essentially, a person harboring the ALDH2*2 allele may not become a heavy drinker. Indeed, genetic epidemiologic studies have indicated that the ALDH2*2 allele inhibits the development of alcoholism. However, many studies demonstrated that patients harboring ALDH2*2 allele who are heavy drinkers were at increased risk of ESCC. It is unknown why patients harboring the ALDH2*2 allele became heavy drinkers despite the unpleasant reaction to acetaldehyde [28-31].

In the present study, heavy drinkers with *ALDH*2*2/2*2, *ALDH*2*1/2*2, and *ALDH*2*1/2*1 genotypes comprised 3.9%, 25.5%, and 14.7% of the cancer group, and 2.5%, 20.0%, and 0.8% of the control group, respectively. It is clear that persons harboring the *ALDH*2*2/2*2 genotype are less likely to be heavy drinkers than those harboring the *ALDH*2*1/2*2 or *ALDH*2*1/2*1 genotypes. Aside from the increased sensitivity of alcohol-induced nausea, this may reflect a very low level of alcohol consumption in Feicheng County, where living standards are relatively low and the majority of farmers cannot afford alcoholic beverages [32,33].

The TT genotype of the *MTHFR *gene had a significantly positive association with ESCC (OR = 1.85, 95% CI 1.02-3.34) but not with BCH or ESCD. There was also a significant association between esophageal cancer and the *MTHFR *TT genotype with which the patient was also a heavy smoker. Associational studies linking polymorphisms of the *MTHFR *C677T genotype with ESCC risk have yielded inconsistent result. A meta-analysis of studies examined the association of the *MTHFR *C667T polymorphism with risk of esophageal cancer [34].

The association between esophageal cancer and *MTHFR *enzyme activity is most likely related to the metabolism of folic acid, as risk of esophageal cancer depends on the status of folic acid intake. When folic acid intake is sufficient, individuals with the *MTHFR *CT or TT genotypes may actually have a decreased risk of esophageal lesions because the lower *MTHFR *activity of the 677TT allele may lead to an elevation in 5, 10-methylenetetrahydrofolate, facilitating DNA synthesis. In contrast, both impaired DNA methylation and DNA synthesis/repair may become the primary mechanisms of carcinogenesis in the presence of low folic acid intake [16,35-37]. However, the TT genotype was not related to BCH or ESCD, suggesting a weaker or absent linkage.

We present evidence that two susceptibility genes, ALDH2*2 and *MTHFR *677T, contribute to the process of esophageal carcinogenesis. The combination of the two high-risk genotypes 2*2/1*2 of *ALDH *2 and TT/TC of *MTHFR *C677T resulted in a 4-fold higher risk of developing BCH, a 3.7-fold increased risk of ESCD, and a 8.72 times higher ESSC risk. The *ALDH *2 and *MTHFR *C677T genes showed a significant association with ESCC in our population.

In contrast to *ALDH *2 and *MTHFR*, polymorphisms of *CYP2E1 *G1259C, *MPO *G463A, and *hOGG1 *C326G genes were not associated with BCH, ESCD, or ESCC risk in this study. These negative results may be attributable to the fact that the study population came from the same community where residents share a similar life style and diet. This homogeneity may cause an over-match, such that the association of these two metabolic enzyme genes (*CYP2E1, MPO*) and one repair gene (*hOGG1*) with lesions of the esophagus cannot be demonstrated or is too low to estimate. In addition, because these alleles were associated with smaller ORs (<2.0) for risk of the diseases, this effects would not be detected due to an allele null frequency less than 0.10. The sample size, therefore, may not have been large enough to detect an association.

## Conclusion

The ALDH2*2 and *MTHFR *677T alleles were associated with higher susceptibility to esophageal cancer. Compared with the *ALDH *1*1 genotype, the *ALHD *2*2 genotype was associated with significantly increased risks for BCH, ESCD, and ESCC, while the TT genotype of *MTHFR *C677T increased the risk of ESCC. The generalized odds ratio analysis confirmed these findings. Further analysis revealed that the combination of these high-risk genotypes (2*2/1*2 of *ALDH *2 and TT/TC of *MTHFR *C677T) significantly increased susceptibility for BCH, ESCD, and ESSC (by 4.0, 3.7 and 8.72 fold, respectively). The OR_G _of the two genotypes combined was 1.83(95%CI: 1.55-2.16), indicating a significant genetic association between this combined genotype and cancer of the esophagus.

## Abbreviations

BCH: Basal cell hyperplasia; ESCD: Esophageal squamous cell dysplasia; ESCC: Esophageal squamous cell cancer; LGD: Low-grade dysplasia; HGD: High-grade dysplasia; *ALDH2*: Aldehyde dehydrogenase-2; *MTFHR*: Methylenetetrahydrofolate reductase; *CYP2E1*: Cytochrome P450 2E1; *MPO*: Myeloperoxidase; *hOGG1*: Human 8-oxoguanine glycosylase 1; OR_G_: generalized odds ratio.

## Competing interests

The authors declare that they have no competing interests.

## Authors' contributions

QDL, HL and MSW carried out study design, literature research, experimental studies, data acquisition, statistical analysis and manuscript preparation (they made the same contribution to the study). TYD, ZYZ, QXF, FYY and QHL participated in literature research, data analysis and manuscript editing. All authors read and approved the final manuscript.

## Pre-publication history

The pre-publication history for this paper can be accessed here:

http://www.biomedcentral.com/1471-230X/11/74/prepub
